# Radial Arterial Lines Have a Higher Failure Rate than Femoral

**DOI:** 10.5811/westjem.2017.11.34727

**Published:** 2018-02-20

**Authors:** Matthew R. Greer, Scott Carney, Rick A. McPheeters, Phillip Aguiniga, Stephanie Rubio, Jason Lee

**Affiliations:** * Kern Medical, Bakersfield, Department of Emergency Medicine, Bakersfield, California; † Kern Medical, Emergency Medicine Assistant Program, Bakersfield, California

## Abstract

**Introduction:**

Arterial lines are important for monitoring critically ill patients. They are placed most commonly in either femoral or radial sites, though there is little evidence to guide site preference.

**Methods:**

This is an ambispective, observational, cohort study to determine variance in failure rates between femoral and radial arterial lines. This study took place from 2012 to 2016 and included all arterial lines placed in adult patients at a single institution. Causes of line failure were defined as inaccuracy, blockage, site issue, or accidental removal. The primary outcome was line failure by location. Secondary outcomes included time to failure and cause of failure.

**Results:**

We evaluated 272 arterial lines over both arms of the study. Fifty-eight lines eventually failed (21.32%). Femoral lines failed less often in both retrospective (5.36% vs 30.71%) and prospective (5.41% vs. 25.64%) arms. The absolute risk reduction of line failure in the femoral site was 20.2% (95% confidence interval [3.7 – 36.2%]). Failures occurred sooner in radial sites compared to femoral. Infection was not a significant cause of removal in our femoral cohort.

**Conclusion:**

Femoral arterial lines fail much less often then radial arterial lines. If placed preferentially in the femoral artery, one line failure would be prevented for every fourth line.

## INTRODUCTION

Arterial lines are important for monitoring and providing care to critically ill patients. Not only do they allow for rapid access to blood, but they also allow a provider continuous access to the patient’s blood pressure, which enables minute titration of vasoactive medications. Traditionally there are two locations for arterial line placement: femoral and radial arteries. The choice between sites is often made according to the provider’s preference with very little evidence guiding this decision.^1^ Although initial beliefs that arterial lines are immune to infection are certainly unfounded,^2^,^3^,^4^ there is evidence that the infection risk is proportionally similar to their central venous counterparts regarding location.^5^,^6^

New evidence has shown that femoral, central venous catheter infection risk is likely overestimated in the modern era of sterile placement and surveillance.^7^,^8^ It has also been shown repeatedly that central arterial monitoring provides different information from both peripheral and non-invasive monitoring.^9^,^10^,^11^ Older studies have shown that the femoral artery is superior to the radial artery for blood pressure monitoring, but these results come from a different era of medicine when placement technique was different and the landscape of monitoring was not what it is today.^12^ It is therefore important to reinvestigate femoral artery access in today’s environment. Line failure adds significant and unnecessary costs to the treatment of critically ill patients, including financial costs (supplies), time (additional procedure), and health (risk of additional procedure to patient as well as time without critical monitoring). In the present study, we attempt to determine if one site is more prone to failure.

## METHODS

We performed an ambispective, observational, cohort study to determine variance in failure rates between femoral and radial arterial lines. This study took place at a single center, a county teaching hospital with 12 adult ICU beds, and was approved by our institutional review board. Any patient with an arterial line placed anywhere in our hospital (in the intensive care unit [ICU], emergency department [ED], and operating rooms) met our inclusion criteria. Providers at our site were not using ultrasound for arterial line placement routinely, so this metric was not evaluated.

Although the specific indication for arterial line placement was not captured in our study, it is customary at our institution to place arterial lines for either ongoing titration of vasopressor agents or expected repeated evaluation of the management of patients with ventilatory support. Our institution uses the Arrow RA-04020 quick kit for radial arterial lines, which is a 20-gauge, 4.25 cm catheter. The Arrow select kit (ASK-04510-UMP) is used for femoral lines, which is also a 20-gauge catheter, though 12 cm in length. All patients in our study were admitted to an ICU bed and were therefore of high acuity. Exclusion criteria were patient age < 18 years old and line removal before 24 hours.

We performed the retrospective arm of this study using the hospital billing database. Records from every patient who received and was successfully billed for an arterial line between January 2012 and June 2015 in our hospital were included. Research assistants (RAs), who were blinded to the study hypothesis (though educated on what an arterial line is), were provided a training presentation on how to extract relevant information from the electronic health record (EHR), including patient’s age, line insertion time, line removal time, and whether line removal was due to failure. We compiled their results into a database, and a pilot quality inprovement study was initially performed on every 20th patient in the study. The two principal investigators then reviewed the data to ensure that data acquisition was accurate between all RAs, demonstrating reliable inter-observer agreement regarding insertion and removal dates and classification of line failure. After confirming that our proposed method of data acquisition was precise, the RAs performed the complete review on the total cohort and the acquired data was kept in a spreadsheet without analysis until the prospective portion of the study was completed.

The prospective arm of the study took place from June 2015 to March 2016. RAs obtained information on every adult patient in whom an arterial line was placed in our hospital during the enrollment period. To ensure capture of all patients, RAs would observe each ICU bed and ED resuscitation bay for new arterial lines three times daily. They compiled an ongoing list of known lines, noting the time of insertion, location of the line (radial vs. femoral), patient age, and patient comorbidities. If the arterial line was found to have been removed, the RAs would document the time of removal and determine why the line had been removed (or if the patient had died), noting whether it was considered a failure and if it was replaced. The RAs obtained this information from nursing flow sheets or nursing interview at the time of their evaluation. Causes of failure included the following: 1) inaccuracy (if a patient was still on vasoactive medications or there was signal dampening or a large discrepancy between noninvasive blood pressure cuff and arterial line), 2) blockage (line would not draw or ABGs still routinely drawn at the time of removal), 3) site issue (hematoma, swelling, concern for infection or neuropathy), and 4) accidental removal (as documented by nursing).

**Population Health Research Capsule**

What do we already know about this issue?

Arterial lines are placed predominantly in the radial and femoral arteries with little known about the site selection effect on patient outcomes.

What was the research question?

Is there a difference in failure rates between arterial lines placed in the radial artery compared with the femoral artery?

What was the major finding of the study?

Femoral Arterial lines failed at a significantly reduced rate as compared to their radial counterparts.

How does this improve population health?

If arterial monitoring is expected for significant amount of time then choosing a site which is less prone to failure leads to improved monitoring and less need for line replacement.

We hypothesized a 2× greater failure rate of radial arterial lines compared to femoral amounting to a 50% reduction in failure rate by placing the line in the femoral artery. We postulated a 60% radial and 40% femoral distribution of line placement, based on observance of local practice. We calculated that 128 patients would provide sufficient power to detect the hypothesized failure rate if lines were split evenly between the two sites. We therefore planned to enroll 200 patients as the actual distribution was not known a priori. We chose an ambispective design as the EHR made retrospective data acquisition easy, allowing for greater power to the study. We subsequently used the prospective data to help validate our retrospective findings.

## RESULTS

**Figure 1:**
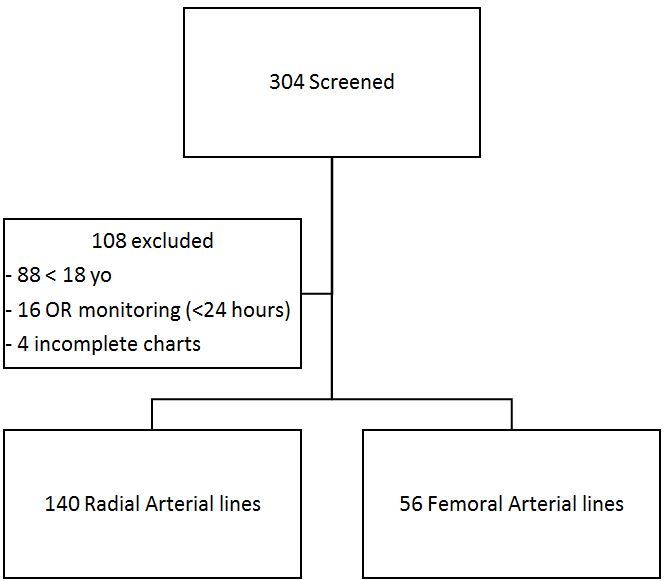
Retrospective patient selection for comparison of radial vs. femoral arterial lines.

**Figure 2:**
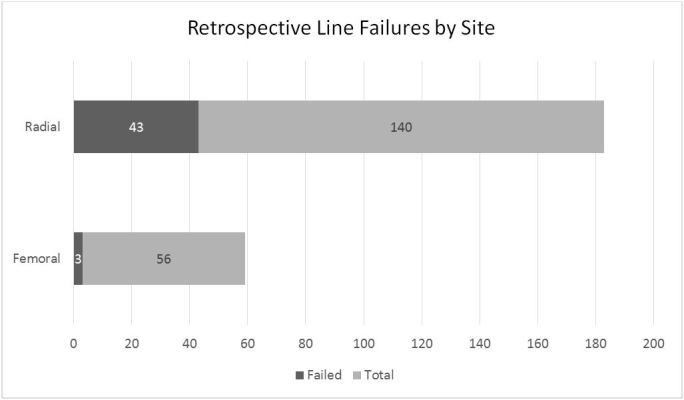
Arterial line failure by site from retrospective data. Femoral lines failed 5.36% of the time (3 of 56) as compared to radial lines, which failed 30.17% of the time (43 of 140).

In total, we evaluated 272 arterial lines over both the prospective and retrospective arms of our study, with 58 lines leading to failure for a combined total failure rate of 21.32%. Comorbidities between the two cohorts were similar, as shown in the Table. Our retrospective arm screened 304 arterial lines; however, only 196 (140 radial and 56 femoral) met criteria for analysis over the three-and-a-half years (Figure 1). The radial cohort had 43 failures (30.71%) and the femoral cohort had three failures (5.36%), for an absolute risk reduction for failure of 25.4% (95% CI [13.7 – 34.2%]) if the femoral site was chosen (Figure 2).

**Figure 3:**
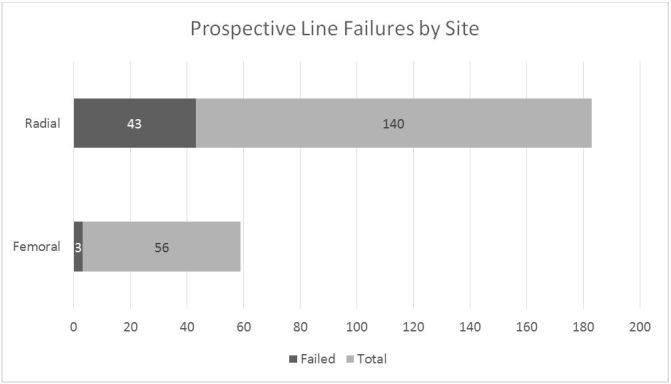
Arterial line failure by site from prospective data. Femoral lines failed 5.41% of time (2 of 37) as compared to radial lines, which failed 25.64% of the time (10 of 39).

The prospective arm had 76 total lines, which included 39 radial and 37 femoral. The radial cohort had 10 failures (25.64%) and the femoral cohort had two failures (5.41%) (Figure 3). This similarly provided an absolute risk reduction of 20.2% (95% confidence interval [CI] [3.7 – 36.2%]) in failure rate if a femoral line was placed instead of a radial arterial line. This outcome was consistent between the retrospective and prospective arms of the trial and led to a number needed to treat (preference of femoral line over radial) of 4.1 patients to prevent one line failure.

Secondary outcomes evaluated include time to failure and cause of failure. Combined data showed the median time to failure for radial lines as two days compared to femoral lines having a median time to failure of four days. From the prospective data, the primary causes of failure for the radial lines were accidental removal (40%), a line not drawing (30%), and inaccurate readings (30%). There were no radial lines removed due to “site issue” (which would include infection) in our prospective arm; however, such issues were responsible for 15% of radial removals in our retrospective arm. Conversely, accidental removal accounted for only 5% of all removals in the retrospective cohort of radial lines but 40% of failures in the prospective arm. In the femoral cohort, site issues and inaccuracies were the causes of two of the removals, and inability to draw was the cause for removal in a single patient. Accidental removal did not occur in the femoral cohort.

Mortality data was only monitored in the prospective arm and included only patients with an arterial line in place at the time of death or patients who died shortly after line removal as we did not follow patients beyond line removal. There were 11 deaths in the femoral cohort, with none occurring in patients with prior femoral line failures. There were seven deaths in the radial cohort, with three of those occurring in patients with prior radial line failures. Of the three deaths attributed to patients with radial line failures, one of these failed lines was replaced in the other radial artery (failed due to inaccuracies), one was replaced in a femoral artery (failed due to accidental removal), and one was not replaced (failed due to accidental removal).

## DISCUSSION

**Figure 4:**
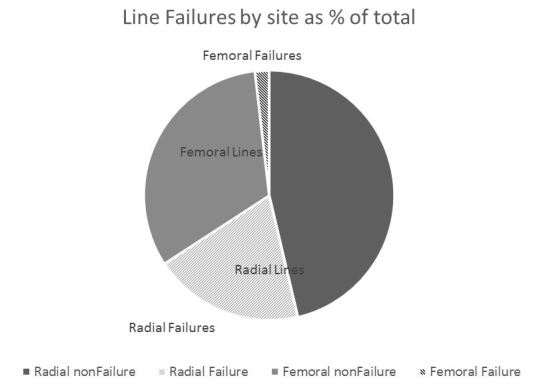
Arterial line failure as % of total, with hashed areas representing failure rates. Femoral failure rates are proportionally much smaller than their radial counterparts.

To our knowledge, this is the first study to compare failure rates by arterial line site in the last 35 years. Soderstrom et al. in 1982 also showed differences in “placement duration” and “longevity” when comparing arterial line sites, with results favoring the femoral site. Their data was similar to ours in that it showed a femoral failure rate of 10.6% compared to 26.4% for radial sites, with failures occurring on average 3.5 days sooner in their radial cohort.^12^ Our data likewise demonstrate that femoral arterial lines fail at a significantly lower rate than radial arterial lines (5.38% compared to 29.61%). Despite making up 34% of the lines placed, they accounted for only 8.6% of the lines that failed (Figure 4). This difference was consistent in both the retrospective and prospective arms of the study, exhibiting agreement between the two data groups. This gives a number needed to treat of only four patients: four patients preferentially receiving a femoral arterial line prevents one premature line failure.

We believe that no meaningful conclusion can be drawn from the data regarding mortality, as we did not follow patients beyond removal of their arterial lines. Additionally, the comorbidity data is likely incomplete given the low prevalence of classical diseases in this critically ill population. This is likely due to limitations of our retrospective review, including incomplete charting and a lack of emphasis on this data during collection.

This study was also not designed to evaluate the reason for a provider’s site preference. At the institutional level, radial lines are preferred over femoral, and this likely instilled selection bias, though to what extent is unclear. If radial lines are preferred by default, then femoral lines might have been placed in cases where there were radial site issues or in patients with higher acuity. Femoral lines might also have been placed after multiple radial attempts failed, or even after a placed radial line failed in the first 24 hours, which would not have been caught by our study (as lines less than 24 hours were excluded). These factors should have selected for a sicker population in the femoral cohort, though this did not lead to increased line failure rates. An alternative argument could be made that because the femoral cohort were sicker, they may have gotten more attention by nursing staff and thus better line care (Hawthorne effect). The possibility of this effect is mitigated, however, by the prospective arm: in this phase nursing staff was aware of the study and yet the line failure rates remained consistent to the retrospective data in each site.

The length of time in which the lines failed also favors femoral line placement. Of the failed arterial lines, the average times of failure for the femoral and radial cohorts were four and two days after placement, respectively. Still, not all radial lines in our study failed within two days, with some radial lines lasting 17 days; however, it is impossible to know which lines are going to fail in advance, and our data indicates that radial lines on average fail earlier in the course.

Although we would have liked to draw conclusions as to why lines failed, our study with only 58 total failures was not adequately powered to draw meaningful conclusions in this area. The ambispective design also makes it difficult to compare causes of failure between the two groups due to the ambiguity of etiology in the retrospective charting. We attempted to account for this in the prospective arm by having the RAs determine the cause of removal from the nursing staff that had direct care of the patient. Our data between the two groups is comparable in this respect, adding strength to our retrospective methods. Furthermore, the length difference between the arterial lines could have an effect on line longevity, but this does not change the fact that femoral lines failed less often. Longer, wider catheters in the radial artery are likely to increase complications, given the increasing risk for ischemia at this site.

Our study suggests that femoral lines were well tolerated with minimal infectious risk. Only one of 93 femoral arterial lines were removed for “site issues”. Note that in designing this study, we wanted to be broad in our attribution of “infection” and thus specified a broader category of “site issue” (rather than “infection”) as a cause of line failure. In retrospect, it would be favorable to define why this one patient’s line was removed (e.g., was it due to hematoma, infection, or some other cause?). This particular line failed after six days and was replaced with another femoral line.

Patients who died early in their disease course may not have had enough time for the line to fail, and this could have led to dilution of the failure rates among sites. Additionally, lines that were not adequate at insertion or were tenuous would increase the number of failures inappropriately as they were likely to get replaced quickly. The retrospective cohort is at greatest risk of being affected by these confounders as providers were unlikely to add additional billing codes for lines replaced rapidly in the same day. We attempted to control for both issues by only including lines surviving greater than 24 hours. The prospective data matching the retrospective rates also gives confidence that we monitored true failure rates.

## LIMITATIONS

This was a single-center study performed at a county teaching hospital with only 12 adult ICU beds, which partially accounts for the small volume of patients over the four-year period of the study. Additionally, our retrospective arm relies on a billing database, which certainly does not capture all lines placed during this period. Several factors would contribute to loss of capture, though we suspect, based on observed practice, that lack of appropriate billing code assignment and documentation of placement in individual procedure notes accounted for the majority of lost lines in the database. The prospective arm remedies this in that RAs observed every bed that might have held a patient with an arterial line three times daily. We believe this is why there were 76 patients captured in the nine months of the prospective arm (8.4 patients/month), and only 196 patients captured in the 42 months of the retrospective arm (4.6 patients/month). The external validity of our study may be limited due to the size of our hospital and ICU; however, we would argue that these are in fact the locations where line failures can be the most detrimental, straining a system already stretched thin.

Given the observational nature of our study, we were not immune to selection bias and it is impossible to determine why one site was chosen over another site by each individual provider. We noticed that more often, patients were given a femoral line as a rescue from a radial line failure, and thus the lines may have been placed at times when the patient’s illness was further along and possibly improving. However this still would not account for the extended time to failure seen in the femoral cohort. Line site preference did change (from 28% femoral placement during the retrospective arm of the study to 49% femoral placement during the prospective arm), which may be due to a change in local culture, a change in perception of femoral line risk given new literature, more complete capture of all lines placed, or even a Hawthorne effect (though this effect is unlikely as many line placements in this portion of the study were performed by providers unaware of the study). It is also likely that many lines were not captured in our retrospective arm as stated above. A better understanding of the differences between the patient groups (demographics, diagnosis, and true mortality rates), as well as the reasons why a site was chosen, was not captured in our study but would be interesting to study in another, larger trial.

## CONCLUSION

These data show that femoral lines fail far less often than radial arterial lines and when they do fail, it occurs later in the patient’s treatment. Further study should endeavor to confirm these findings across multiple centers and practice styles, including at larger institutions and with the use of ultrasound for placement. If it is in fact determined that arterial line failure rates could be reduced from almost 30% to 5% simply by privileging femoral over radial sites, that would lead to significant gains in patient care in terms of less time unmonitored, less exposure to risk, and lower cost to the healthcare system.

## Figures and Tables

**Table:** Comorbidities across cohorts in a study comparing failure rates by arterial line site

	Radial	(%)	Femoral	(%)	P
		
Comorbidities	N = 179	-	N = 93	-	
Alcohol use	21	(11.73%)	10	(10.75%)	0.810
Chronic kidney disease	4	(2.23%)	5	(5.38%)	0.171
Congestive heart failure	9	(5.03%)	5	(5.38%)	0.904
Coronary artery disease	11	(6.15%)	7	(7.53%)	0.667
Diabetes	32	(17.88%)	15	(16.13%)	0.712
Hypertension	46	(25.70%)	22	(23.66%)	0.711
